# Supplementary material for: Phase Coexistence of Mn Trimer Clusters and Antiferromagnetic Mn Islands on Ir(111)
Source: ACS Nano. 2024 Jan 16;18(4):3699–706. doi: 10.1021/acsnano.3c11459 (PMC10832046; doi:10.1021/acsnano.3c11459)
Supplement: Supplementary file 1 — nn3c11459_si_001.pdf [file nn3c11459_si_001.pdf]

Supplementary information for:

Phase Coexistence of Mn Trimer Clusters and Antiferromagnetic Mn Islands on Ir (111)

Arturo Rodríguez-Sota*, Vishesh Saxena, Jonas Spethmann, Roland Wiesendanger, Roberto Lo Conte§, André Kubetzka, Kirsten von Bergmann#

Institute for Nanostructure and Solid State Physics, University of Hamburg, 20355 Hamburg, Germany

* arturo.rodriguez@physnet.uni-hamburg.de

# kirsten.von.bergmann@physnet.uni-hamburg.de

§ Present address: Zernike Institute for Advanced Materials, University of Groningen, 9747 AG Groningen, The Netherlands

Table of contents:

Supplementary figure 1- STM images for different Mn coverages and preparation temperatures**.**

Supplementary figure 2- Models for the reconstruction.

Supplementary figure 3- Cluster pair correlation and cluster density.

Supplementary figure 4- Cluster evolution and stability while imaging.

Supplementary figure 5- Dissecting a Mn trimer using atom manipulation.

Supplementary figure 6- STM measurements at different temperatures.

Supplementary figure 7- Extreme low coverage sample.

Supplementary figure 8- Proto-Islands.

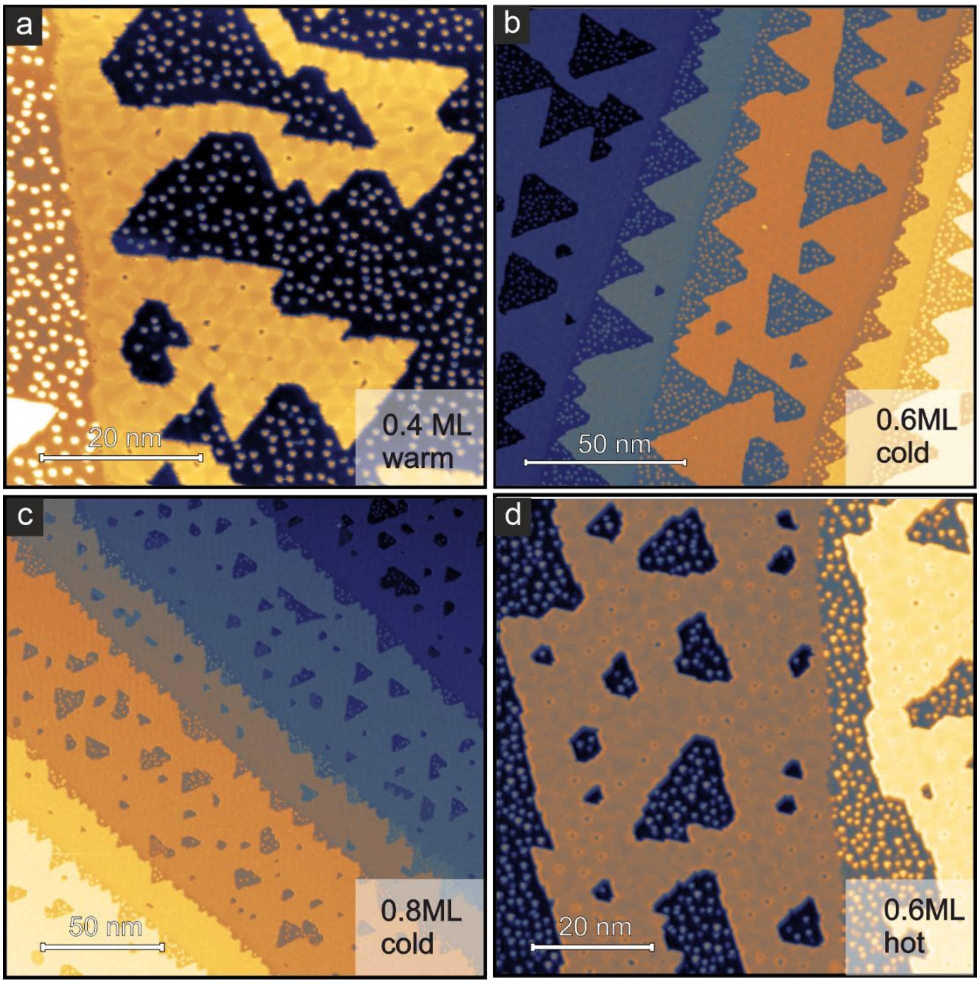

Supplementary figure 1- STM images for different Mn coverages and preparation temperatures: (a) Bias voltage U = 50 mV, tunnel current I = 1 nA, measurement temperature T = 80 K, time between flash and evaporation t = 20 min, (b) U = 100 mV, I = 500 pA, T = 4 K, t = 45 min (c) U = 800 mV, I = 1 nA, T = 8 K, t = 53 min (d) U = -400 mV, I = 500 pA, T = 4 K, t = 8 min.

Figures **a** and **c** show two extreme cases of sub-monolayer coverages, 40% and 80% of a ML respectively, for which the clusters have statistically been studied; note that here the coverage refers to the surface area covered by the reconstructed Mn monolayer. In **a** the island coverage is 40% of a ML, one of the lowest ones studied. The Mn evaporation was performed 20 minutes after flashing, resulting in a warm sample. In **c** the island coverage is 80% of a ML, one of the highest coverages where vacancy islands allow to study the clusters. The growth was done 53 minutes after the flashing, giving rise to a room temperature substrate. The figures **b** and **d** show two different samples prepared with the same island coverage of 60% of a ML. The Mn deposition in **b** was started 45 minutes after flashing, whereas in **d** the time between the last flash and Mn deposition was only 8 minutes and therefore the sample temperature is expected to be higher compared to the preparation shown in **b** due to the time needed by samples to return to room temperature. In conclusion, we find a coexistence of clusters and islands independent of the island coverage and the deposition temperature when evaporating the Mn.

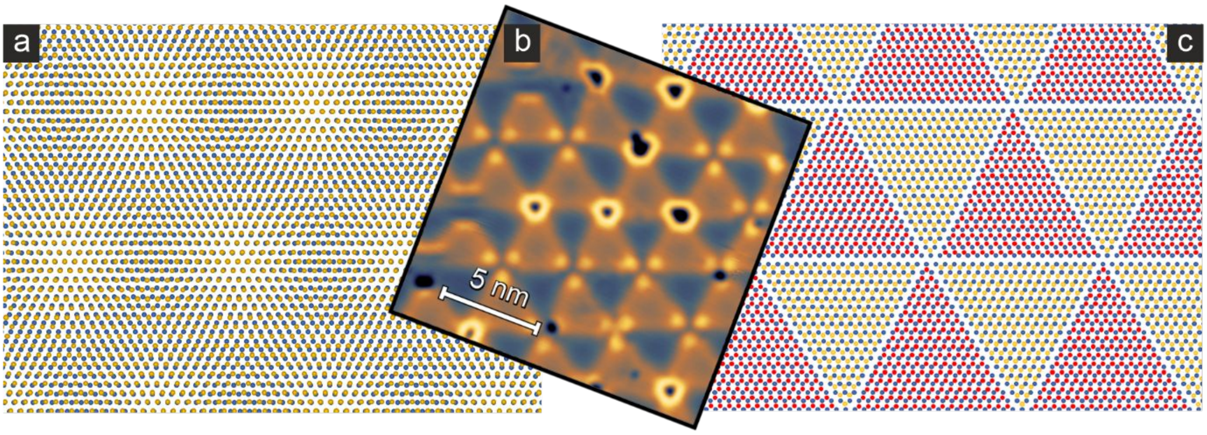

Supplementary figure 2- Models for the reconstruction: (a) Moiré model generated by the superposition of a hexagonal top layer expanded by 7% with respect to the bottom one. (b) STM constant-current STM image of the reconstructed first monolayer of Mn on Ir (111) (U = 10 mV, I = 1 nA, T = 4 K), (c) Tessellation pattern consisting of differently stacked triangular islands.

For the reconstruction of the Mn islands, we propose two different models: Figure **a** shows a Moiré-like structure with a Mn top layer that is expanded by 7% in comparison to the underlying Ir(111) surface due to lattice strain. The 7% expansion is obtained comparing the Néel state within the reconstructed Mn monolayer with the Néel state in the pseudomorphic Mn monolayer (main text figure 2). Figure **c** shows a structure composed of discrete triangular patches with different stacking types, i.e., hcp and fcc stacking. On one hand the moiré model explains the observed stacking changes and the common appearance of holes at the corners of the triangles. These positions correspond to the on-top positions in the moiré pattern, which are expected to be energetically unfavoured. On the other hand, the discrete model helps understanding the abrupt change in stacking and the clear differentiation between pointing up and pointing down triangles that is observed in STM images. In reality we suspect that the actual structure is a mixture of these two models, i.e. a moiré pattern as shown in figure **a** but with sharper transitions in between the two stacking types.

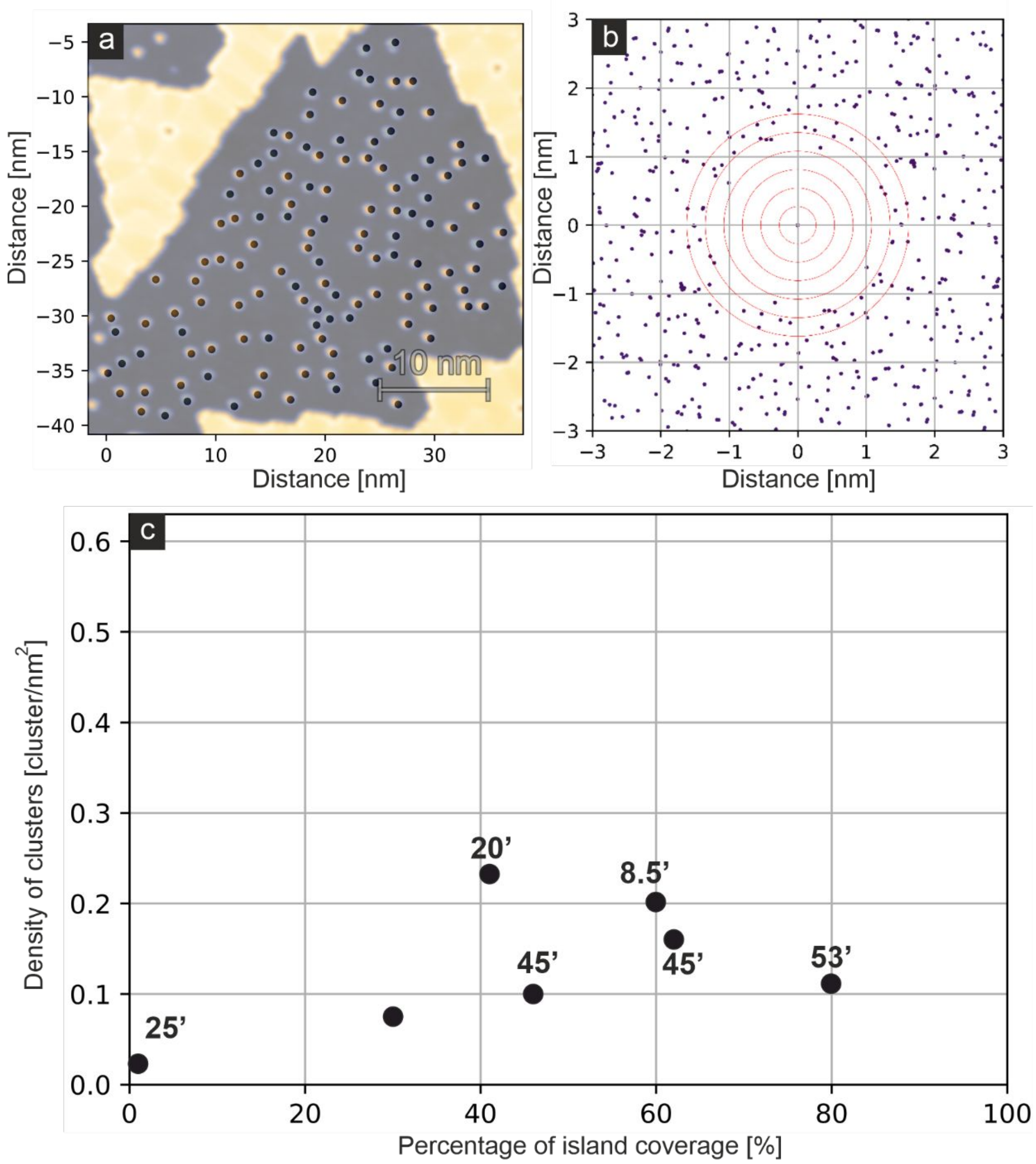

Supplementary figure 3- Cluster pair correlation and cluster density: Data analysis of cluster distribution used to identify potential superstructures in the cluster positioning. (a) Real-space position of trimers in a particular sample superimposed on the original STM image from which the data was taken, $U = 50$mV, $I = 1$nA, $T = 4$K. (b) Pair correlation of said positions indicated in panel a. (c) Density of clusters depending on the island coverage for several preparations. Numbers next to data points indicate the time in minutes between flash and Mn deposition.

Image **a** shows the experimentally observed positions of clusters in a vacancy island. Image **b** shows the pair correlation of said trimers. This represents the real space relation of every cluster with all the other clusters. The red circles superimposed on the image **b** are separated by one atomic distance between them. The only observed interaction between trimers is a short distance repulsion, as they do not appear closer than five atomic distances (1.35 nm) from each other.

The image **c** shows the density of clusters dependence on the island coverage. The temperature of the sample during the Mn deposition is related to the time passed after the last flash (indicated in the figure). The cluster density typically varies between 0.1 and 0.2 clusters/nm^2 and we observe no clear relation between the cluster density and the deposition temperature or the overall Mn island coverage.

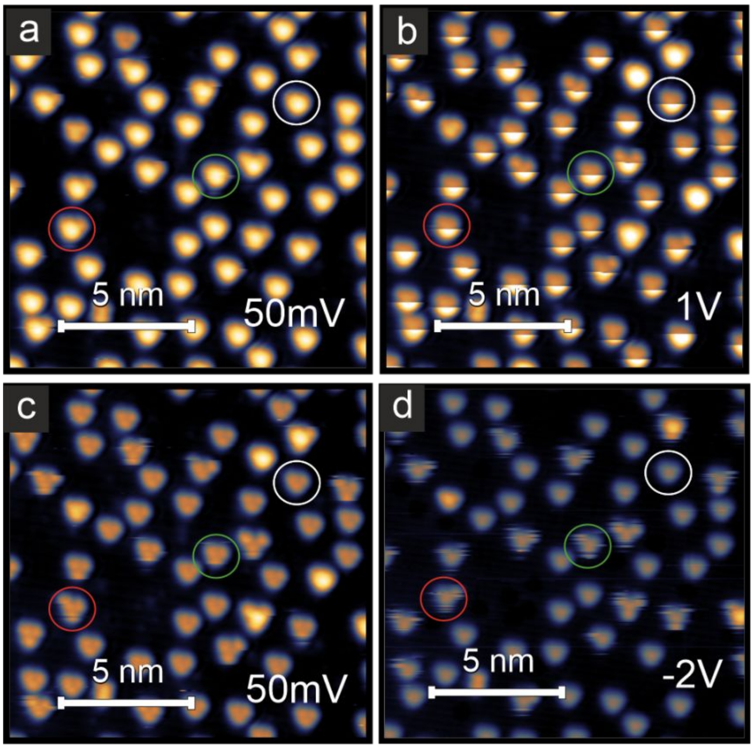

Supplementary figure 4- Cluster evolution and stability while imaging: (a)-(d) Series of constant-current STM measurements showing how high clusters can be converted to low clusters when measuring with certain bias voltages ($T = 80$ K). Very high bias voltages (image d) help demonstrating the stability of the low clusters. (Measurement parameters: **a** $U = +50$ mV, $I = 100$ pA, **b** $U = +1$ V, $I = 4$ nA, **c** $U = +50$ mV, $I = 100$ pA, **d** $U = -2$ V, I = 4 nA.)

This series of STM measurements shows how acquiring images with a high bias voltage can trigger a change in the cluster structure. Image **a** was taken before the experiment to show the original state of the clusters. Image **b** shows the exact moment in which each cluster transforms from a high cluster to a low cluster when the tip scans over them. Image **c** shows the same area after most of the clusters have been switched measured with the same voltage and represented in the same scale as image **a**. Image **d** was measured with an even higher bias voltage than image **b** ($U = 2$ V) and allows us to identify stable and unstable low/diluted clusters based on their movement when applying said voltage.

Analysing the Mn clusters, we observe three different cases in the change from high to low:

1.- Compact trimers that evolve to stable dilute trimers (white circle)

2.- Compact trimers that evolve to unstable dilute trimers (green circle)

3.- Asymmetric trimers that evolve to unstable dilute trimers (red circle)

In all cases, high clusters evolve to the same apparent object, the dilute trimer, but the two different types of behaviours of the seemingly equal objects (stable and unstable dilute trimers) indicate that they are indeed different species of trimers. This was later used to identify the two stackings and eventually create the cluster model (see Fig. 4**c** in the main text).

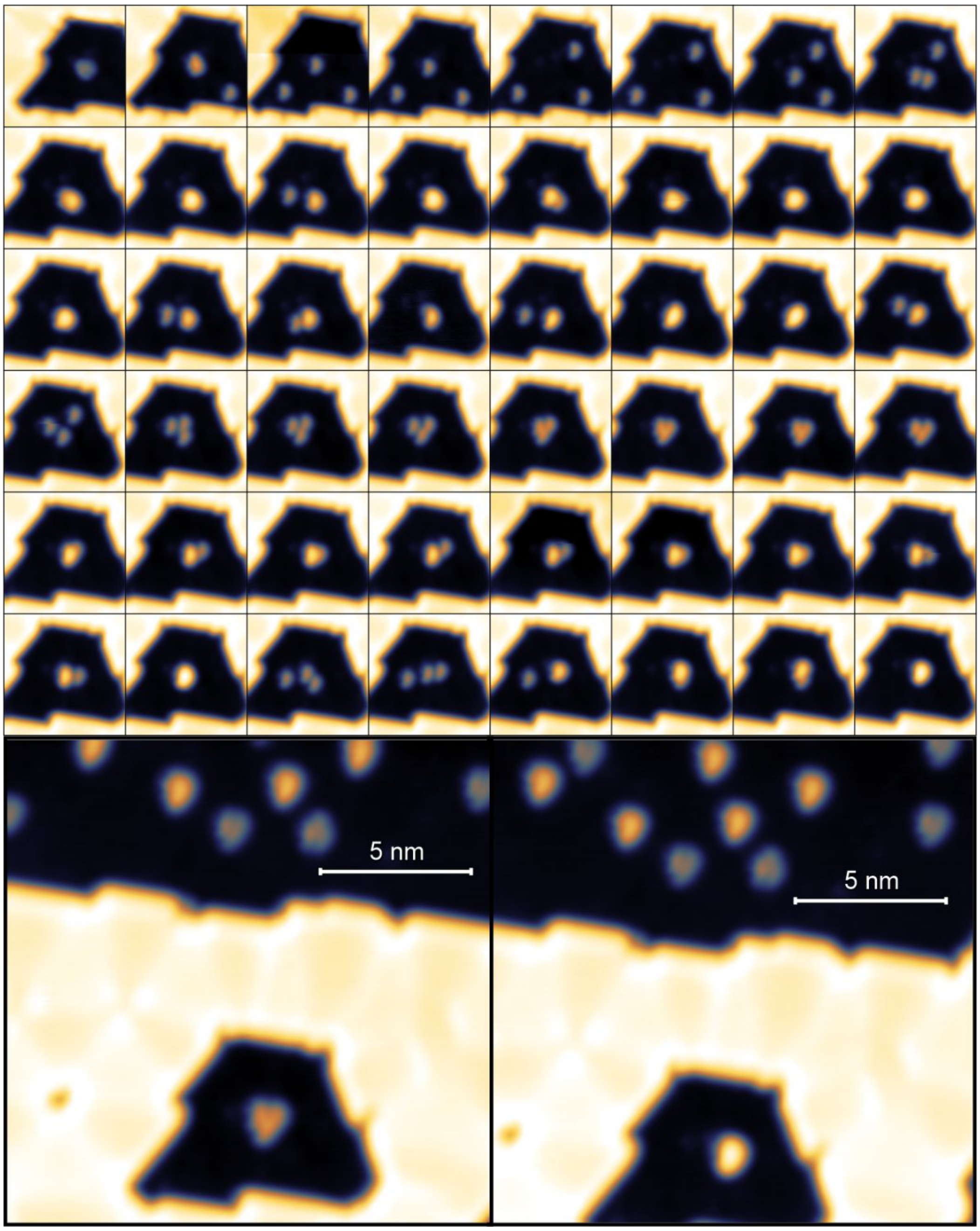

Supplementary figure 5- Dissecting a Mn trimer using atom manipulation: Series of STM images taken between atom manipulation experiments dissecting a cluster in a vacancy island. Typical parameters for the images are $U = 5$ mV and $I = 2$ nA. The atom manipulation in between images was performed with parameters varying from 1-5 mV and 70-90 nA.

This series of STM images shows the deconstruction and reconstruction of a Mn cluster on the Ir(111) surface. First the cluster was separated into three individual atoms and then these atoms were used to create a new cluster. This procedure was repeated several times resulting in different clusters. Their configurations among them give rise to the different types of trimers that have been observed. The last two bigger figures at the end show “handmade” trimers inside the vacancy island in comparison with other natural trimers.

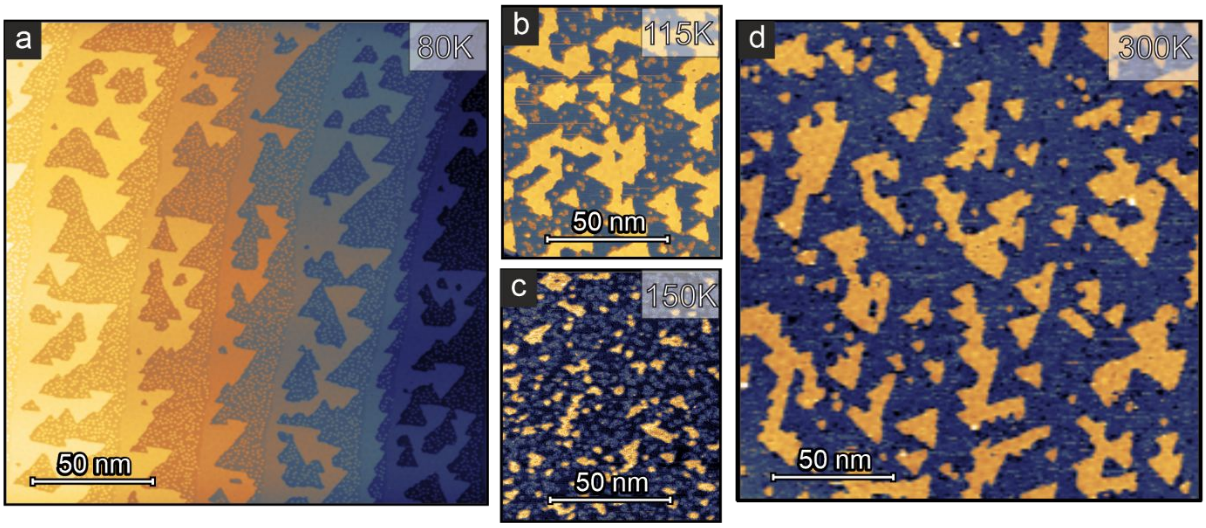

Supplementary figure 6- STM measurements at different temperatures. Constant-current STM images measured at different temperatures. Measurement parameters: **a** $U = 50$mV, $I = 100$pA, $T = 80$K, **b** $U = 650$mV, $I = 600$pA, $T = 115$K, **c** $U = 6$mV, $I = 50$pA, $T = 150$K, **d** $U = 650$mV, $I = 800$pA, $T = 300$K.

Images **a** to **d** show the appearance of samples studied at different temperatures by STM. In figure **a**, at 80 K the presence of clusters is observed. In figure **b**, at 115 K, most of the time clusters look unidentifiable as they seem to have merged with each other, but are still distinguishable from the Mn islands and the Ir substrate. In figure **c**, at 150 K, clusters are no longer identifiable as such and the cluster model is no longer applicable. Clusters appear constantly unstable and can no longer be defined as individual entities. In figure **d**, at 300K, clusters are no longer visible. We suspect that they move on the Ir substrate freely. We think the clusters cannot be observed at this temperature because the speed at which they move over the Ir surface is much faster than the data acquisition time of the STM. Their occasional movement between tip and sample results in an unstable tunnel junction when scanning on top of the Ir.

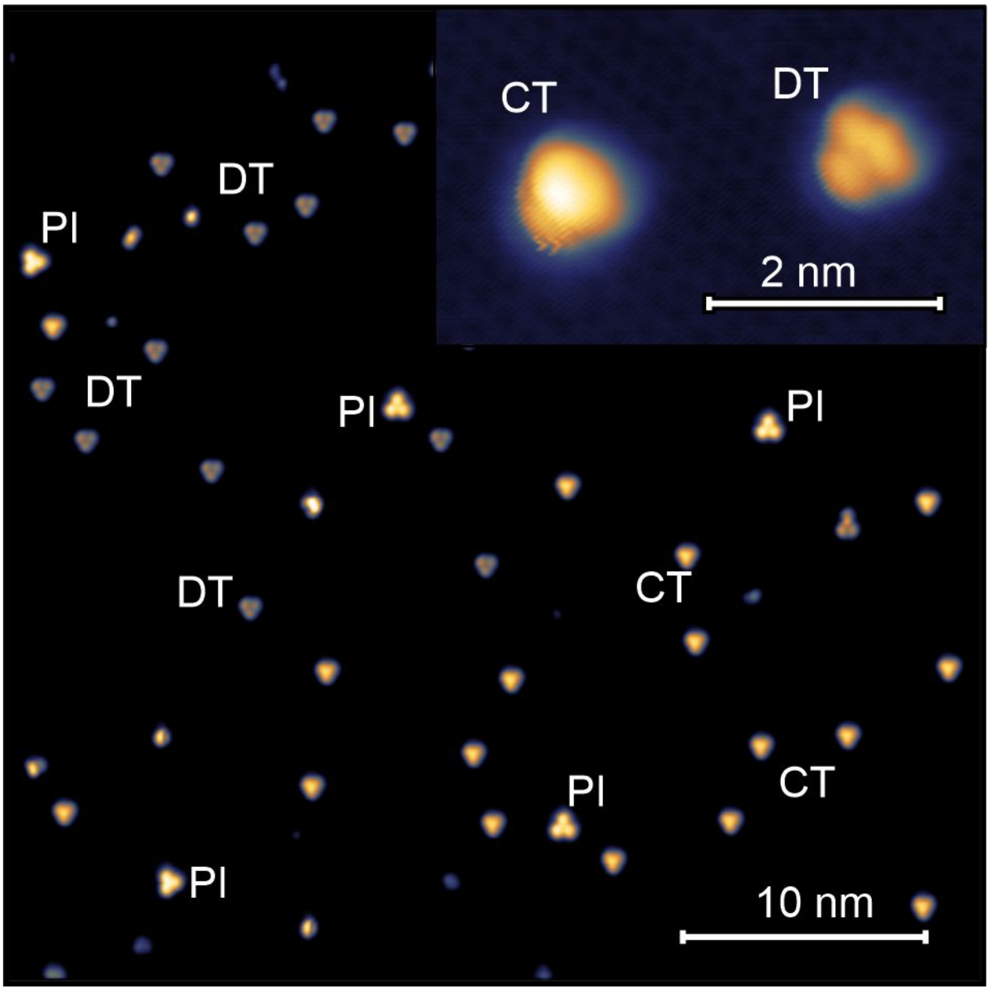

Supplementary figure 7- Extreme low coverage sample: Overview STM image of a sample evaporated with Mn for 2 seconds (on the order of 0.05 monolayers). Measurement parameters: $U = 3$ mV, $I = 1$ nA, $T = 4$ K, inset, $U = 20$ mV, $I = 1$ nA, $T = 4$ K. Compact trimers are indicated as CT, diluted trimers as DT and Proto-Islands as PI.

This figure shows the Ir(111) surface after two second Mn evaporation. This corresponds to an expected coverage of 0.05 monolayers. Islands are not observed at all in this coverage regime, which indicates that a certain density of clusters seems to be needed for their growth. The cluster density is about 0.024 clusters/nm^2, i.e., much lower as compared to the density when clusters and islands coexist. Nevertheless, the majority of clusters consists of three atoms, demonstrating their unique stability. They behave as the previously observed trimers, including the change from tall to short when high voltages are applied. In addition to the trimers another type of structure appears in the sample: They are triangular three-lobed objects of bigger size and height than the clusters. We call these structures *proto-islands (PI),* as we suspect that they are the precursors of islands in their smallest nucleation stage. We attribute the existence of these proto-islands to the presence of defects behaving as nucleation centres.

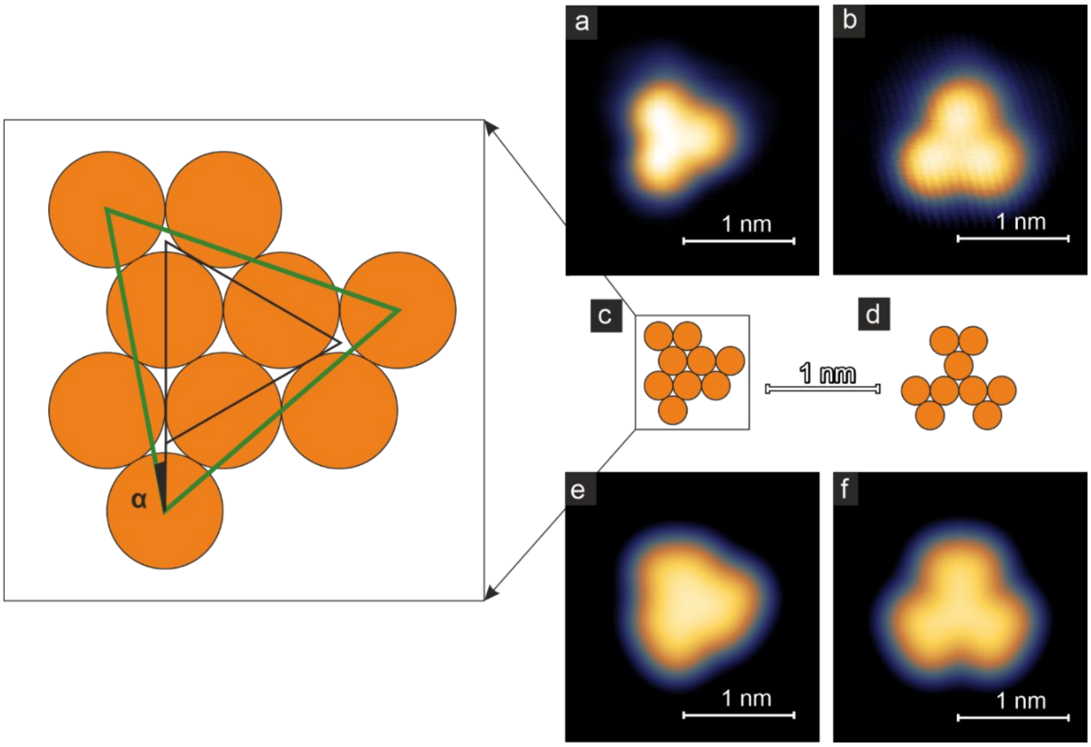

Supplementary figure 8- Proto-Islands: (a), (b) Constant-current STM images of two different proto-islands (measurement parameters: **a**: $U = 3$ mV, **b**: $U = 5$ mV, **a,b**: $I = 1$ nA, $T = 4$ K). (c), (d) Proposed atomic structure models for the proto-islands in **a** and **b**. (e), (f) Constant-height STM simulations of the atomic structures in **c** and **d**. Black lines in the inset show the triangle formed by connecting the centres of the three constituting trimers, whereas the green triangle is formed by connecting the outermost atoms of the proto-island. The offset angle between these two triangles is indicated by α.

Images **S9.a** and **S9.b** show two types of proto-islands that can be distinguished because they appear as triangles pointing in different crystallographic directions. Proto-islands like the one in figure **a** seem to have just one maximum in the centre, in contrast, the proto-island shown in figure **b** has three well defined maxima (one on each lobe). The areal ratio between these two types of proto-islands is b/a = 1.15.

The proposed model in **c** and **d** comes from the idea that at room temperature clusters are mobile and that proto-islands are formed by an agglomeration of these trimers. Based on these assumptions, the structural models in **c** and **d** were created in an attempt to match the experimentally observed proto-islands. The more compact structure of the proto-islands in **c** explains why only one contrast maximum can be observed for the proto-island in **a**, whereas the more open structure of the proto-island shown in **d** explains why there are three distinguishable maxima for this type of proto-island.

To validate our atomic structure model, we compared the experimental data with the simulations. The areal ratio between the two simulated proto-islands is 1.11, an error of about 3.6% with respect to the experimental value. In addition, we observe that the proto-islands as shown in **a** appear to be slightly rotated with respect to the crystallographic high-symmetry directions. We define this rotation as the angle α (see inset of figure **S9.c**). This rotation can be explained in the model if we understand that the triangular shape observed in the STM data is generated by the outermost atoms of each proto-island forming, i.e., by the green triangle instead of the smaller black triangle. In the experiment we measure an angle of $\alpha = 13.5°$, whereas from the simulated image we extract an angle of $\alpha = 13.26°$. Thus, the experimental and simulated data are in good agreement.
